# Supplementary material for: Depth-dependent peridotite-melt interaction and the origin of variable silica in the cratonic mantle
Source: Nat Commun. 2021 Feb 17;12:1082. doi: 10.1038/s41467-021-21343-9 (PMC7889928; doi:10.1038/s41467-021-21343-9)
Supplement: Supplementary file 1 — Supplementary Information [file 41467_2021_21343_MOESM1_ESM.pdf]

Methods

Details of natural data compilation

To assess the global pattern of MgO/SiO_2 in Archaean cratonic lithosphere, we carefully compiled comprehensive SCLM peridotite data from the literature. From this, we extracted bulk-rock major element compositions for garnet-bearing and -free low temperature, undeformed peridotites from the six cratons for which sufficient data are available (Kaapvaal, Siberia, Slave, Rae, Tanzania and North Atlantic cratons). Where no bulk-rock major element compositions were available, we calculated them from reported phase compositions and phase abundances determined by point-counting or image analysis. Only coarse-grained peridotites (olivine >40 %) were included, whereas porphyroclastic varieties (using the terminology of Harte[1] were not considered The dataset was filtered to remove samples showing evidence for FeO addition, disequilibrium and alteration according to the following criteria:

1) olivine and/or whole rock must have Mg# >89.3 (i.e. greater than in pyrolite mantle [2] and whole rock ≥8.05 wt% FeO_T;
2) where mineral data are available, olivine Mg# and orthopyroxene Mg# must be correlated; and
3) abundances of the main minerals (olivine, orthopyroxene, clinopyroxene and garnet/spinel) must total >90% (where listed separately, serpentine was added to the olivine mode).

Modal abundances were normalised to 100% on a phlogopite-free basis and then classified using the USGS ultramafic scheme. The resulting peridotites include only dunite, harzburgite and lherzolite. The full dataset is provided as supplementary information.

THERMOCALC modelling

The files necessary for performing the calculations may be found by following links to software at https://www.esc.cam.ac.uk/directory/tim-holland

Modeling the compositions of residues after fertile peridotite melting

Phase equilibrium modeling was performed using THERMOCALC version tc350beta[3] with the internally consistent thermodynamic dataset of Holland and Powell[4] (update tc-ds634) in the system KNCFMASTOCr (K_2O-Na_2O-CaO-FeO-MgO-Al_2O_3-SiO_2-TiO_2-Fe_2O_3-Cr_2O_3) using activity–composition models for peridotite-forming minerals (garnet, spinel, clinopyroxene, orthopyroxene, and olivine) and

melt[5], which are based on the peridotite model of Jennings and Holland[6]. As well as incorporating Na_2O and TiO_2, the updated model includes improvements to the position of the orthopyroxene-out boundary in fertile peridotites, resulting in more accurate reproduction of the modal abundance of orthopyroxene above the solidus. We used pyrolite-like composition KR4003[7] as the starting composition to investigate melting of fertile peridotite mantle and calculated the residue compositions from isobaric batch melting at 1-6 GPa and at 2% increments up to 60% melting or orthopyroxene/garnet out, which ever was later. The ferric iron content of the natural peridotites was set to 0.3 wt% Fe_2O_3 ($Fe^{3+}/\Sigma Fe=0.03$). It should be noted that the phase equilibrium model does not distinguish between a single event of high degree melting and the physically much more plausible scenario of multiple melting events that collectively reach a high degree of melting. We use Mg# (Mg/Mg+Fe) as a proxy for the degree of partial melting. The modeled residue compositions were found to be consistent with experimentally determined residues from partial melting of fertile peridotite in the range 1-6 GPa (Fig. S1).

Modelling melt-rock reaction

Melt-rock reaction modeling was undertaken using THERMOCALC version tc350beta. The starting compositions for batch melting-reaction modeling were mixtures of fertile mantle composition KR4003[7] and moderately and highly depleted peridotite. For low pressure basalt-peridotite reactions, we used DMM1[8] and the melting residue from T-4243[9], for komatiite-peridotite reaction, we used the residues of melting experiments 60.01 and 60.05[7], these starting compositions span a range of Mg#, CaO and Al_2O_3 expected for peridotite formed by partial melting of fertile peridotite. The melt compositions used for low and high pressure melt-rock reaction, respectively, were either olivine tholeiite with 13.9 wt% MgO produced by 13.5 wt.% melting of fertile peridotite at 2 GPa leaving a lherzolitic residue (run 21[10]; or komatiite with 35 wt.% MgO produced by ~70% melting at 8 GPa leaving olivine and garnet in the residue (run 78[11]. Starting melt and peridotite compositions are listed in table S1. Calculations were performed at 20 and 50% basalt/komatiite, and at 1 GPa increments from 1 to 3 GPa (basalt) and 1 to 6 GPa (komatiite) and were performed 60% melting or to orthopyroxene out, which ever was later.

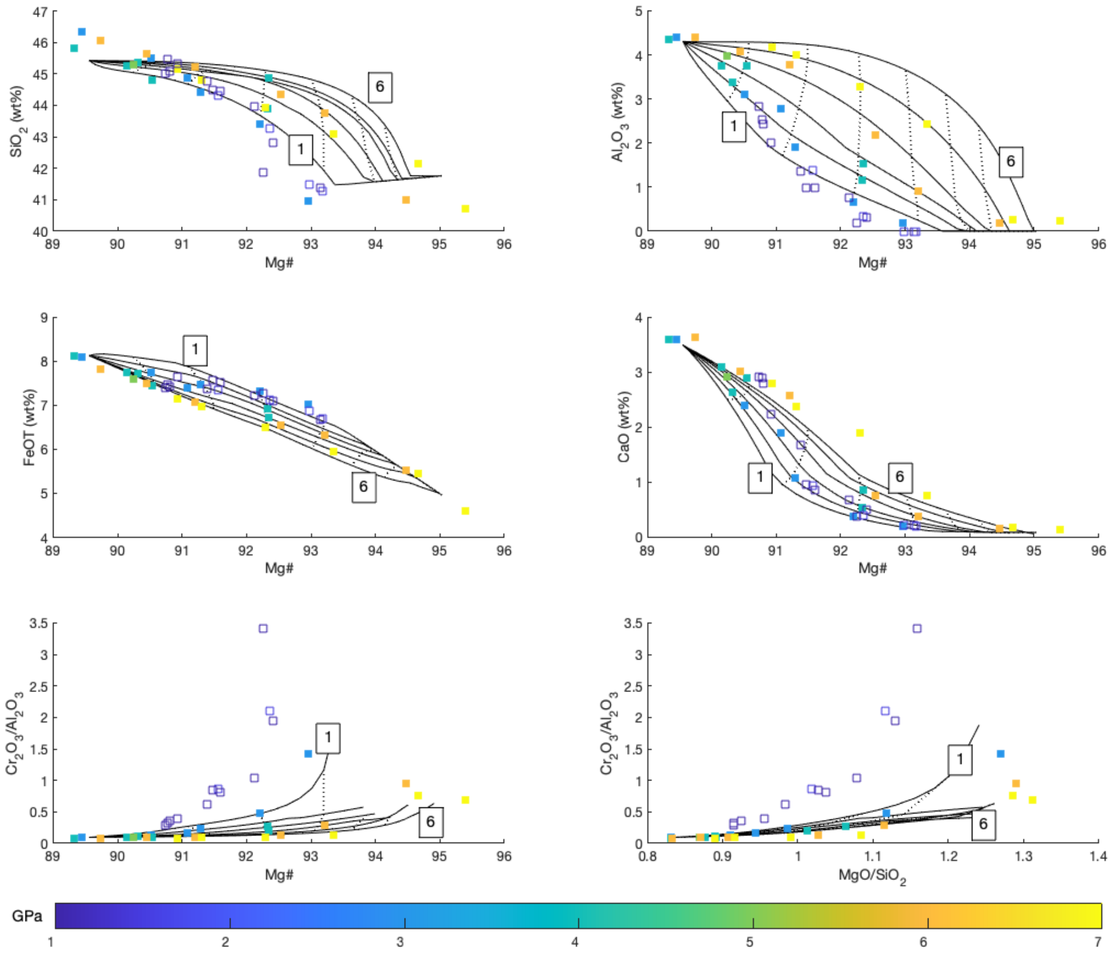

Figure S1: Calculated composition of residue produced after melting of fertile peridotite KR4003 (black lines) in 1 GPa increments from 1 to 6 GPa (minimum and maximum pressures are labelled). The degree of is shown as dashed lines increments of F = 0.1. Calculated trends are compared to experimental residues after melting of MM-3[9,12,13] (open symbols) and KR4003[7] (closed symbols) which are coloured to indicate pressure. Note: the Cr_2O_3 content of MM-3 is twice as large as that of KR4003.

Reaction coefficients

The coefficients of the melting reactions were calculated from the slopes of the modelled trends in plots of liquid proportion vs. proportions of crystalline phases in the residue (see Baker and Stolper[12]). Reaction coefficients are tabulated in the supplementary data, spinel is not shown as spinel does not contribute significantly to the melting reactions and reaction coefficients are small

			Peridotite			Reacting melt	
Name	KR4003	DMM1	T-4243	60.01	60.05	Run 21	Run 78
Type	fertile	depleted	depleted	depleted	depleted	Basalt	Komatiite
Ref.	Walter 1998	Wasylenki et al. 2003	Falloon et al. 1999	Walter 1998	Walter 1998	Hirose and Kushiro 1993	Takahashi et al. 1986
	-	Residue of 12-13% melting at 1GPa	Residue of 31% melting at 1GPa	Residue of 11% melting at 6GPa	Residue of 41% melting at 6GPa		
SiO_2`	44.9	44.91	42.92	45.64	44.3	47.47	46.6
TiO_2	0.16	0.04	0.43	0.07	0.02	0.75	0.2
Cr_2O_3	0.41	0.39	0.08*	0.37	0.28	0.21	0.4
Al_2O_3	4.26	2.38	0.30	4.08	2.18	15.53	4.6
FeO	8.02	8.34	7.10	7.49	6.55	8.51	8.8
MgO	37.3	41.49	48.47	39.77	45.55	13.94	34.9
CaO	3.45	2.14	0.51	3.01	0.75	11.11	3.9
Na_2O	0.22	0.055	0.01	0.19	0.03	2.22	0.3
K_2O	0.09	0.006	-	-	-	0.08	-
Mg#	89.2	89.9	92.4	90.4	92.5	74.5	87.6

Table S1: starting peridotite and melt compositions used for modelling melt-rock reaction. *Cr_2O_3 concentration has been halved to correct for the more Cr-rich starting composition of MM-3 (Falloon et al. 1999) relative to KR4003 and KLB-1 (Walter 1998; Takahashi et al. 1986; Hirose and Kushiro, 1993).

KDE plots of Mg# and Ni (wt%) in whole rock cratonic peridotite samples

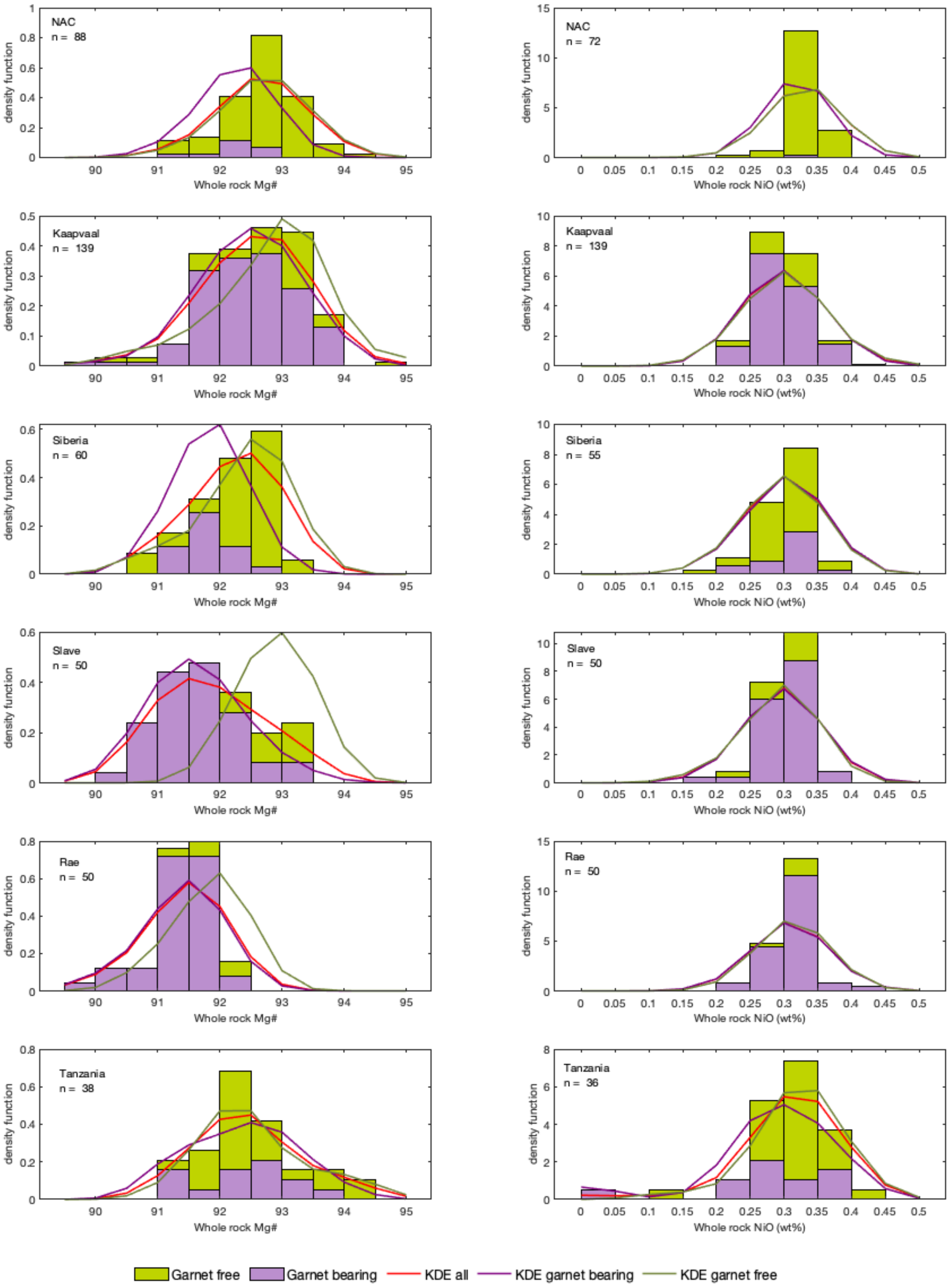

Figure S2: Normalised stacked histograms and Kernel Distribution Estimations of Mg# and NiO in garnet-bearing and garnet-free peridotites from the studied cratons. All locations show a normal distribution of Mg# and NiO, although garnet-free and garnet-bearing peridotites may have different modes.

Reaction of basalt with depleted lithosphere

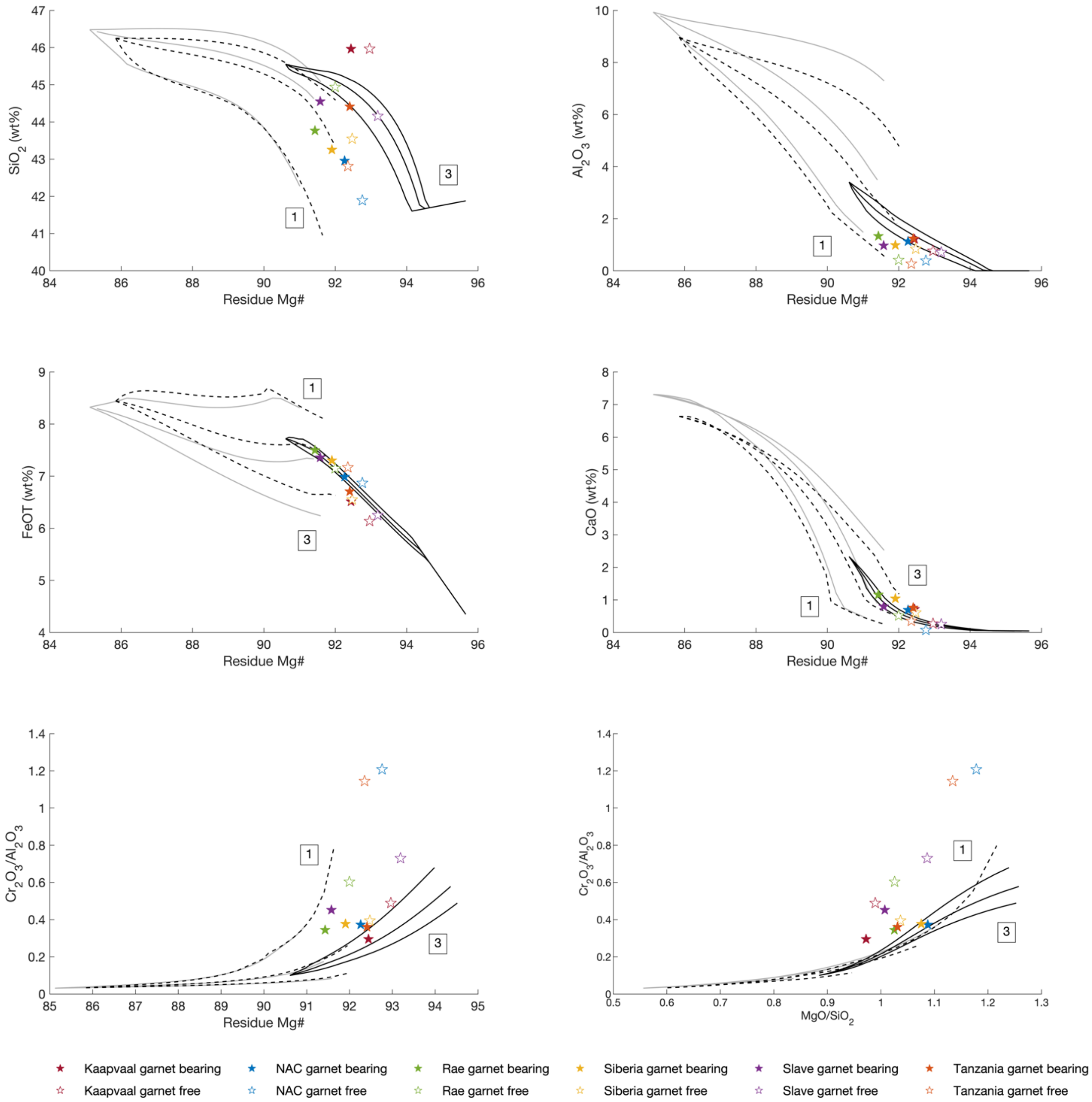

Figure S3: Compositions of residues from melting of hybrid rocks composed of 50% basalt (run 21[10]) and 50% fertile peridotite KR4003[7] with Mg# 89.2 (grey lines), moderately depleted peridotite DMM1[8] with Mg# 89.9 (black dashed lines) and highly depleted peridotite the residue from melting experiment T-4243[9] Mg# 92.4 (solid black lines) modelled using THERMOCALC. Maximum and minimum pressure is indicated in GPa. Median compositions of garnet bearing (solid symbols) and garnet free (open symbols) cratonic peridotites.

Reaction of komatiite with depleted lithosphere

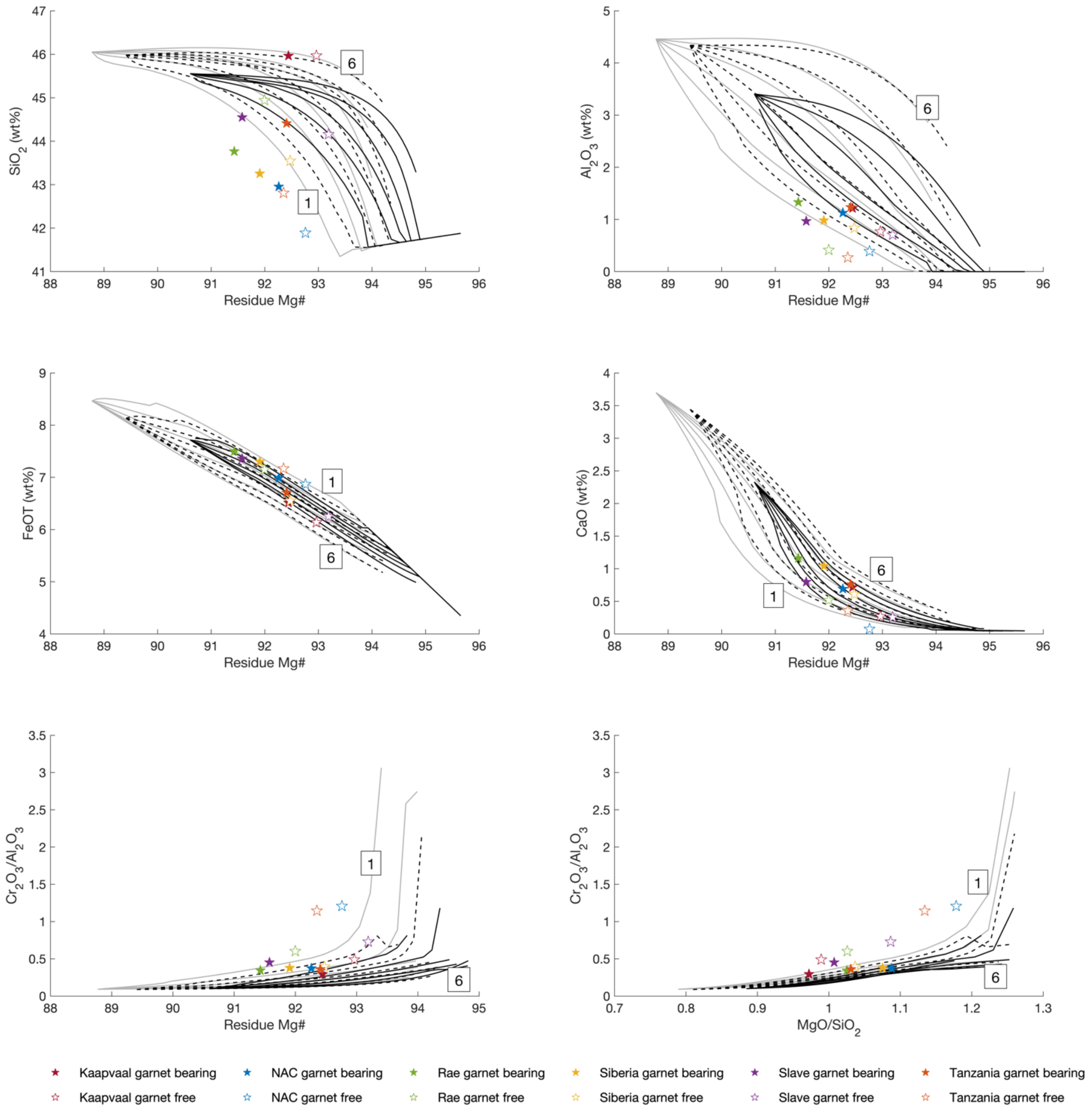

Figure S4: Compositions of residues from melting of hybrid rocks composed of 50% komatiite (run 78[11]) and 50% fertile peridotite KR4003[7] with Mg# 89.2 (grey lines), moderately depleted peridotite with Mg# 90.4 (bulk residue from run 60.01[7]; black dashed lines) and highly depleted peridotite with Mg# 92.5 (bulk residue from run 60.05[7]; solid black lines) modelled using THERMOCALC. Maximum and minimum pressure is indicated in GPa. Median compositions of garnet bearing (solid symbols) and garnet free (open symbols) cratonic peridotites.

References

1. Harte, B. Rock Nomenclature with Particular Relation to Deformation and Recrystallisation Textures in Olivine-Bearing Xenoliths. *J. Geol.* **85**, 279–288 (1977).
2. McDonough, W. F. F. & Sun, S.-S. S. The composition of the Earth. *Chem. Geol.* **120**, 223–253 (1995).
3. Tomlinson, E. L. & Holland, T. J. B. A Thermodynamic model for the subsolidus evolution and melting of peridotite: application to the cratonic lithosphere. *J. Petrol.*
4. Holland, T. J. B. & Powell, R. An improved and extended internally consistent thermodynamic dataset for phases of petrological interest, involving a new equation of state for solids. *J. Metamorph. Geol.* **29**, 333–383 (2011).
5. Holland, T. J. B., Green, E. C. R. & Powell, R. Melting of Peridotites through to Granites: A Simple Thermodynamic Model in the System KNCFMASHTOCr. *J. Petrol.* **59**, 881–900 (2018).
6. Jennings, E. S. & Holland, T. J. B. A Simple Thermodynamic Model for Melting of Peridotite in the System NCFMASOCr. *J. Petrol.* **56**, 869–892 (2015).
7. Walter, M. J. Melting of garnet peridotite and the origin of komatiite and depleted lithosphere. *J. Petrol.* **39**, 29–60 (1998).
8. Wasylenki, L. E., Baker, M. B., Kent, A. J. R. & Stolper, E. M. Near-solidus Melting of the Shallow Upper Mantle: Partial Melting Experiments on Depleted Peridotite. *J. Petrol.* **44**, 1163–1191 (2003).
9. Falloon, T. J., Green, D. H., Danyushevsky, L. V & Faul, U. H. Peridotite melting at 1.0 and 1.5 GPa: an experimental evaluation of techniques using diamond aggregates and mineral mixes for determination of near-solidus melts. *J. Petrol.* **40**, 1343–1375 (1999).
10. Hirose, K. & Kushiro, I. Partial melting of dry peridotites at high pressures: Determination of compositions of melts segregated from peridotite using aggregates of diamond. *Earth Planet. Sci. Lett.* **114**, 477–489 (1993).
11. Takahashi, E. Melting of a dry peridotite KLB-1 up to 14 GPa: Implications on the Origin of peridotitic upper mantle. *J. Geophys. Res.* **91**, 9367–9382 (1986).
12. Baker, M. M. . & Stolper, E. . E. E. . Determining the composition of high-pressure mantle melts using diamond aggregates. *Geochim. Cosmochim. Acta* **58**, 2811–2827 (1994).
13. Baker, M. B., Hirschmann, M. M., Ghiorso, M. S. & Stolper, E. M. Compositions of near-solidus peridotite melts from experiments and thermodynamic calculations. *Nature* **375**, 308–311 (1995).
