# Supplementary material for: Depth-dependent peridotite-melt interaction and the origin of variable silica in the cratonic mantle
Source: Nat Commun. 2021 Feb 17;12:1082. doi: 10.1038/s41467-021-21343-9 (PMC7889928; doi:10.1038/s41467-021-21343-9)
Supplement: Supplementary file 3 — Description of Additional Supplementary Files [file 41467_2021_21343_MOESM3_ESM.pdf]

Description of Additional Supplementary Files

File Name: Supplementary Data 1
Description: Global dataset of modal mineralogies and bulk compositions of cratonic peridotites compiled from the literature.

File Name: Supplementary Data 2
Description: Phase equilibria during melting of pyrolite, pyrolitebasalt and pyrolite/komatiite calculated using THERMOCALC.
